# Supplementary material for: Morganella Phage Mecenats66 Utilizes an Evolutionarily Distinct Subtype of Headful Genome Packaging with a Preferred Packaging Initiation Site
Source: Microorganisms. 2022 Sep 7;10(9):1799. doi: 10.3390/microorganisms10091799 (PMC9503643; doi:10.3390/microorganisms10091799)
Supplement: Supplementary file 1 [file microorganisms-10-01799-s001.zip › microorganisms-1865726-supplementary.pdf]

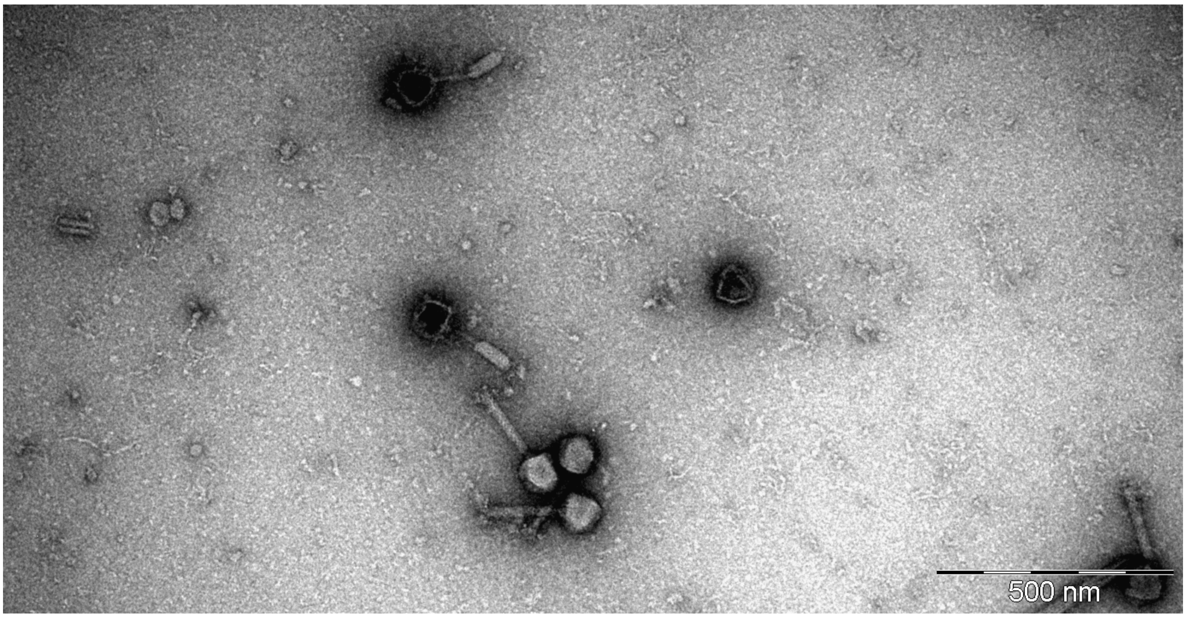

Figure S1: Transmission electron micrograph demonstrating a characteristic field of view for a non-purified phage lysate containing *Morganella* bacteriophage Mecenats66 virions (~10^{10} PFU/mL) negatively stained with 0.5% uranyl acetate. Both intact and defective virions or parts of thereof are visible.

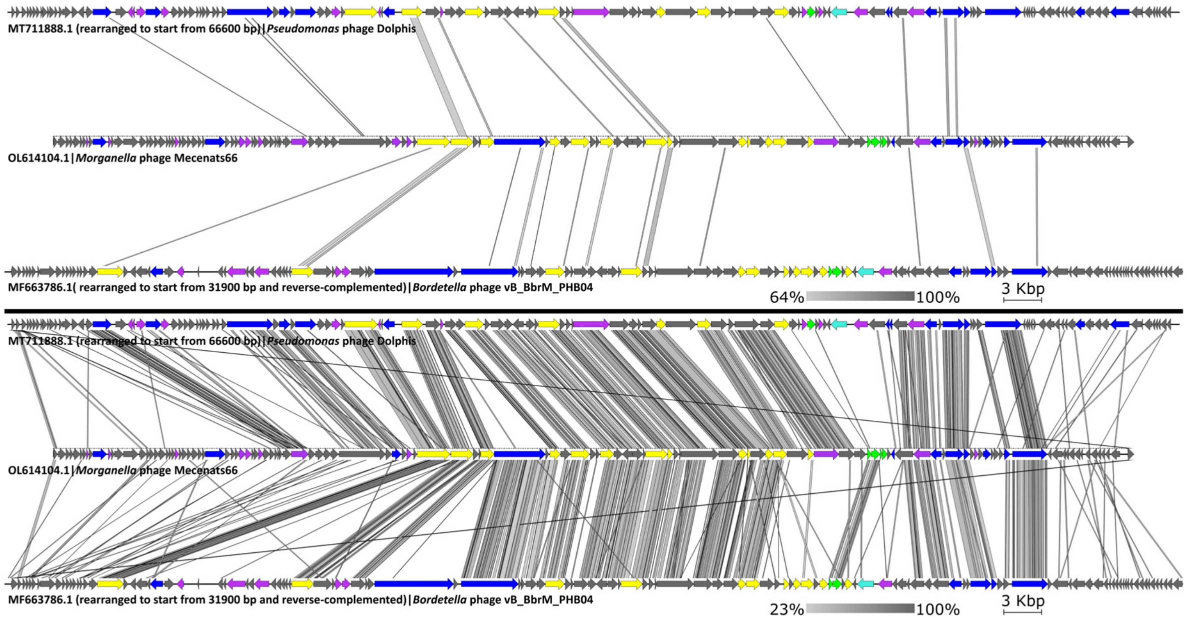

Figure S2: Pairwise genome nucleotide sequence and genome organization comparison of *Morganella* phage Mecenats66 (middle) to *Pseudomonas* phage Dolphis (upper), and to *Bordetella* phage vB_BbrM_PHB04 (lower) using BLASTN (upper tile) and TBLASTX (lower tile). Genomes are drawn to scale, genomes of Dolphis and PHB04 were reordered as indicated to ensure collinearity with Mecenats66; the scale bar indicates 3000 base pairs. Arrows representing the open reading frames point in the direction of transcription and are color-coded based on the function of their putative product: yellow—phage particle morphogenesis, green—lysis, blue—DNA replication, modification, and repair, cyan—lysogeny, purple—additional functions, gray— hypothetical protein of unknown function. Gray boxes represent regions of similarity between the genomes and are colored in a gradient according to their identity; darker shades of gray represent a higher identity. The pairwise comparisons were carried out using EasyFig (v.2.2.2.; [67]).

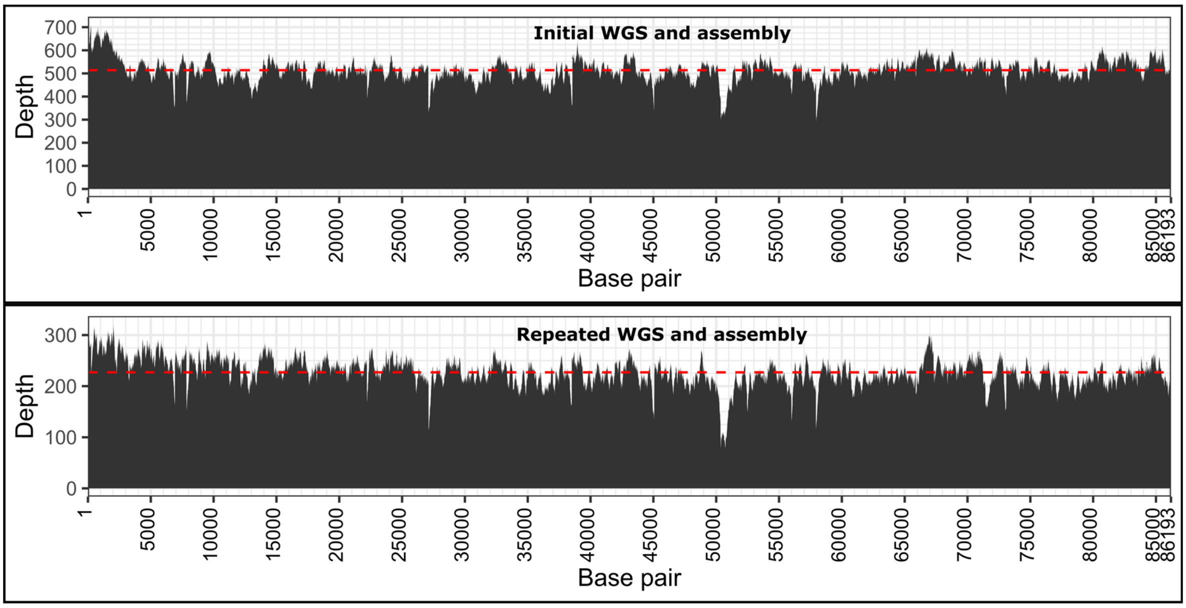

Figure S3: Whole genome base depth histograms of the initial (upper tile) and repeated (lower tile) whole genome sequencing of the *Morganella* bacteriophage Mecenats66 genomic DNA. Corresponding experimental reads were mapped onto the correctly reorganized de novo assembled genome of Mecenats66. Red dashed horizontal lines indicate the mean coverages.
